# N, S-codoped carbon dots for antioxidants and their nanovehicle potential as molecular cargoes†

**DOI:** 10.1039/d4ra05994h

**Published:** 2024-10-10

**Authors:** Md Kasif, Abdullah Alarifi, Mohd Afzal, Arunkumar Thirugnanasambandam

**Affiliations:** a School of Energy Science and Engineering, Indian Institute of Technology Guwahati Guwahati 781039 India; b Department of Chemistry, College of Science, King Saud University Riyadh 11451 Saudi Arabia maslam1@ksu.edu.sa; c Centre for Sustainable Materials and Surface Metamorphosis, Chennai Institute of Technology Chennai Tamilnadu 600069 India arunkumar.t@citchennai.net

## Abstract

This work demonstrates the facile one step hydrothermal synthesis of carbon dots doped with nitrogen and sulfur (SCDs). The carbon dots have various uses, including their use as molecular payloads for antioxidant and drug delivery purposes. The sizes of the CDs were determined using transmission electron microscopy (TEM), which revealed an average size of 4.2 nm. The successful sulfur doping was confirmed by Fourier-transform infrared (FTIR) spectroscopy and X-ray photoelectron spectroscopy (XPS), which identified typical functional groups and elemental composition. UV-vis and photoluminescence (PL) spectroscopy revealed a wide absorption peak at 280 nm and a pronounced blue emission at 440 nm. Colloidal stability was confirmed by dynamic light scattering (DLS) and zeta potential analysis. The antioxidant characteristics were evaluated through the use of electron paramagnetic resonance (EPR) spectroscopy, which confirmed a notable ability to scavenge radicals which revealed more than 80% radical scavenging capability. The SCDs also showed nontoxic behavior against living cells. The findings emphasize the potential of SCDs in the fields of bioimaging, drug delivery, and as potent antioxidant agents.

## Abstract 2

This work demonstrates the facile one step hydrothermal synthesis of carbon dots doped with nitrogen and sulfur (SCDs).

## Graphical abstract

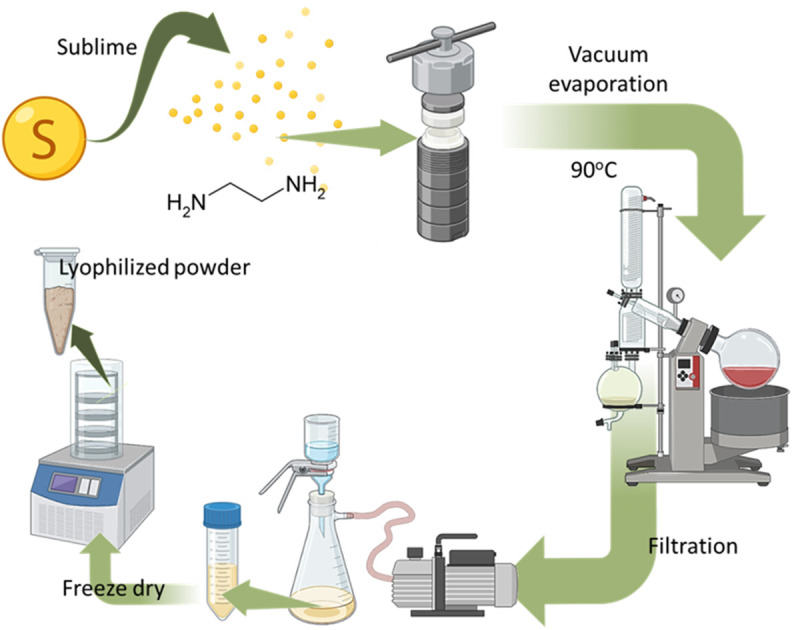

## Introduction

Luminescent nanoparticles, such as semiconductor quantum dots, and silicon quantum dots, are photoluminescent metal compounds that have significantly altered the traditional understanding of photoluminescence in recent years.^1–3^ These nanoparticles possess unique chemical and physical characteristics.^4–6^ Among these, the recently discovered carbon dots (CDs), a unique type of nanocarbon, have gained significant attention in both the scientific community and technical field due to their exceptional photophysical and chemical properties.^7–9^ CDs have attractive features such as low photobleaching, adjustable emission, remarkable hydrophilicity, as well as outstanding biocompatibility, little cytotoxicity, and easy surface decoration.^10,11^ Fluorescent C-dots have a diameter smaller than 10 nm, possess minimal toxicity and exceptional stability.^12^ In addition, the physical and chemical properties have made it possible to create applications by overcoming the limits that are associated with the use of heavy metals, carcinogenic solvents, and high temperatures. Moreover, CDs exhibit a high affinity for both organic and inorganic compounds, facilitated by their surface functional groups. Utilizing these benefits, a range of adaptable synthesis methods and diverse applications were investigated such as photocatalysis, chemical sensing, bioimaging, drug delivery, fluorescent labeling, phototherapies, and more.^13–17^ Until now, CDs have been produced using various precursors. However, researchers are currently searching for appropriate precursors to improve the luminous properties, which are usually suppressed by passivating agents and hazardous compounds. Therefore the synthesis of fluorescent CDs with unique properties using various bottom-up methods, including hydrothermal, solvothermal, microwave irradiation, ultrasonic, and thermal breakdown procedures is significant. In this context, CDs generated from natural or renewable resources are drawing the attention of researchers.^18,19^ Green synthesis facilitates the production of CDs by reducing the use of harmful and cancer-causing solvents.^19^ Fluorescent carbon dots were developed using various natural resources such as crab shells, prawn shells, green tea leaves, coffee, garlic, ginger, and bio wastes as carbon sources.^20,21^ This approach can address the issue of surface passivation.^22^ These materials are chosen for their eco-friendly nature, affordability, therapeutic capabilities, and widespread availability.^23–26^

CDs have recently earned the interest of researchers as a means of delivering medications in order to enhance the availability of drugs in specific organs while minimizing their negative impact on normal tissue.^27,28^ CDs have a large surface area with a sp^2^ core, allowing them to form connections with different hydrophobic proteins by π–π* stacking or electrostatic interactions. This enhances the solubility of these biomolecules in water.^29^ In addition, medicines can form covalent bonds with the functional groups on the surface of CDs.^30^ Additionally, the fluorescent properties of the CDs allow for the tracking of the drug delivery mechanism.^31^ However, there are only a limited number of researches demonstrating the utilization of CDs as a drug delivery mechanism, as the majority of studies have primarily concentrated on investigating their optical features.^32^ Antioxidant behavior is another significant characteristic in biomedical applications, as they are a fundamental factor in the development of illnesses in biological living systems.^33^ The production of free radicals is accountable for the occurrence of these ailments through the alteration of proteins, harm to deoxyribonucleic acid, cancer, and so on. Furthermore, inflammation has become prevalent in contemporary society and is interconnected with chronic conditions through its impact on cell membranes.^34^ Frequent use of traditional treatments might lead to various side effects such as kidney failure and heart attacks. Therefore, this highlights the importance and indispensability of creating an alternative, such as CDs, that has a minimal impact on living organisms.^35^ This led to the development of CDs and their screening for antioxidants and anti-inflammatory compounds through the integration of traditional medicinal plant species and sophisticated nanoscience. Heteroatom functionalization is the predominant approach utilized for modifying CDs. CDs may readily include heteroatoms, such as nitrogen, sulfur, and boron, by utilizing reactants that contain these elements.^36^ Kolekar *et al.* employed a one-step hydrothermal technique to produce blue-emitting sulfur-doped carbon dots (S-CDs) utilizing jaggery as a carbon precursor.^37^ The synthesized carbon quantum dots exhibited minimal toxicity, excellent water solubility, anti-interference characteristics, and persistent fluorescence. The introduction of heteroatoms into CDs aids in reducing toxicity and enhancing functional characteristics, such as antibacterial and antioxidant activities. Heteroatom CDs have been documented to exhibit superior electrocatalytic performance for oxygen reduction compared to homoatomic CDs.

Thus, the objective of this study was to formulate heteroatom co-doped CDs by incorporating nitrogen and sulfur in a single-step hydrothermal method. The prepared CDs have shown excitation dependent fluorescence change, easy aqueous dispersibility, colloidal stability, antioxidant behavior, and excellent nanocarrier for therapeutic payloads. Sulfur doping, in conjunction with nitrogen, creates distinctive redox-active sites that collaboratively augment the antioxidant efficacy of the carbon dots, beyond the performance observed with single-atom doping. This dual doping enhances radical scavenging efficiency, demonstrated by over 80% radical neutralization and EPR spectra, while simultaneously optimizing the electronic structure for superior fluorescence properties. Additionally, the kinetics of drug transport and release over time were assessed in various physiologically simulated fluids. The release trends were examined to formulate a hypothesis regarding the drug-carrying behavior of the SCDs. Additionally, the proposed CDs also have shown non-cytotoxic behavior against living fibroblasts cells which could promote the fluorescent nanodots as an antioxidant nanovector for drug delivery applications.

## Materials

Sublimed sulfur powder and ethylenediamine (C_2_H_4_(NH_2_)_2_) were procured from Sigma-Aldrich, Germany. All other chemicals used were of analytical grade.

### Synthesis of N, S-doped carbon dots (SCDs)

The synthesis of doped CDs was conducted by a hydrothermal technique employing elemental sulfur and ethylenediamine as starting materials. At first, 0.5 g of purified sulfur was dissolved in 50 milliliters of ethylenediamine (H_2_N–CH_2_CH_2_–NH_2_) inside a 100 milliliter Teflon-lined stainless steel autoclave. The mixture was then vigorously stirred for 30 minutes to ensure that it was evenly mixed. The autoclave was thereafter sealed and heated to a temperature of 180 °C in an oven for 12 h, which facilitated the process of carbonization and doping. Following the reaction, the autoclave was cooled to the ambient temperature, and the resultant solution was filtered under vacuum to exclude any residual sulfur or bigger particles, so guaranteeing the purity of the produced SCDs. The filtrate was subsequently concentrated utilizing a rotary evaporator at a temperature of 60 °C while applying reduced pressure in order to decrease the volume to roughly 10 mL. The SCDs solution was freeze-dried by subjecting it to freezing at a temperature of −80 °C and then sublimating the ice under reduced pressure. This process resulted in the formation of a dry and powdered form of SCDs.

### Characterizations

The structure and dimensions of the SCDs were analyzed using a JEOL JEM-2100 transmission electron microscope at an accelerating voltage of 200 kV. The identification of functional groups in the SCDs was carried out using FTIR analysis utilizing a Thermo Scientific Nicolet iS50 FTIR spectrometer. The optical characteristics of the SCDs were assessed using a Shimadzu UV-2600 UV-vis spectrophotometer. Photoluminescence measurements were conducted utilizing a Horiba Jobin Yvon Fluorolog-3 spectrofluorometer. Analyzed utilizing a Kratos Axis Ultra DLD X-ray photoelectron spectrometer with a monochromatic Al Kα X-ray source (1486.6 eV), the elemental composition and chemical states of the SCDs were determined. The XPS survey spectra identified peaks corresponding to the C 1s, N 1s, O 1s, and S 2p, demonstrating the existence of carbon, nitrogen, oxygen, and sulfur in the SCDs. The hydrodynamic size distribution and surface charge of the SCDs in an aqueous solution were determined using a Malvern Zetasizer Nano ZS. The dynamic light scattering (DLS) tests revealed an average hydrodynamic diameter of approximately 10 nm, indicating a minor degree of aggregation in the solution.

## Results and discussions

**Fig. 1:**
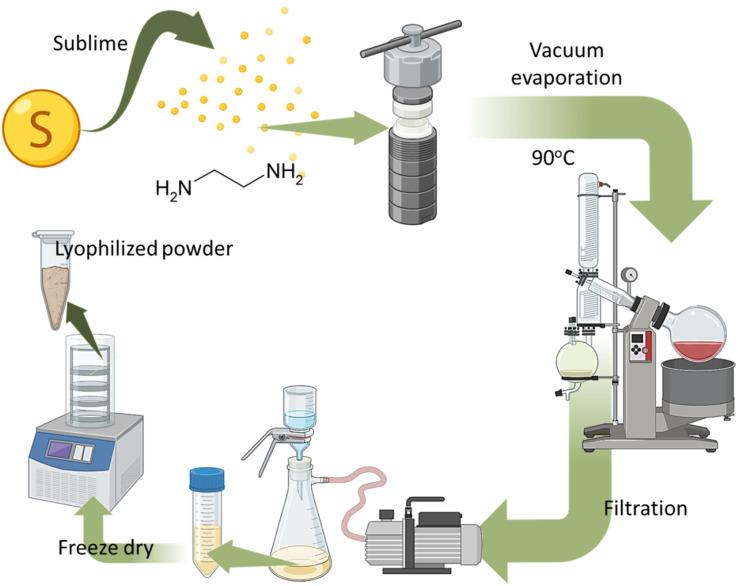
Schematic of S-doped carbon dots by hydrothermal method.

The synthesis of N, S-codoped CDs involved a hydrothermal process utilizing sulfur and ethylenediamine, as shown in Fig. 1. The synthesis technique was meticulously refined to attain superior SCDs with favorable characteristics for prospective biomedical applications. At first, sulfur in its elemental form was evaporated and then mixed with ethylenediamine (H_2_N–CH_2_CH_2_–NH_2_) in a precise ratio of moles to enable sufficient incorporation of sulfur into the CDs structure. Subsequently, the combination underwent a hydrothermal reaction at a temperature of 90 °C for duration of 12 hours. This approach enables the preparation of SCDs by carbonizing and simultaneously doping sulfur and nitrogen atoms into the carbon matrix.

### Physical properties of SCDs

**Fig. 2:**
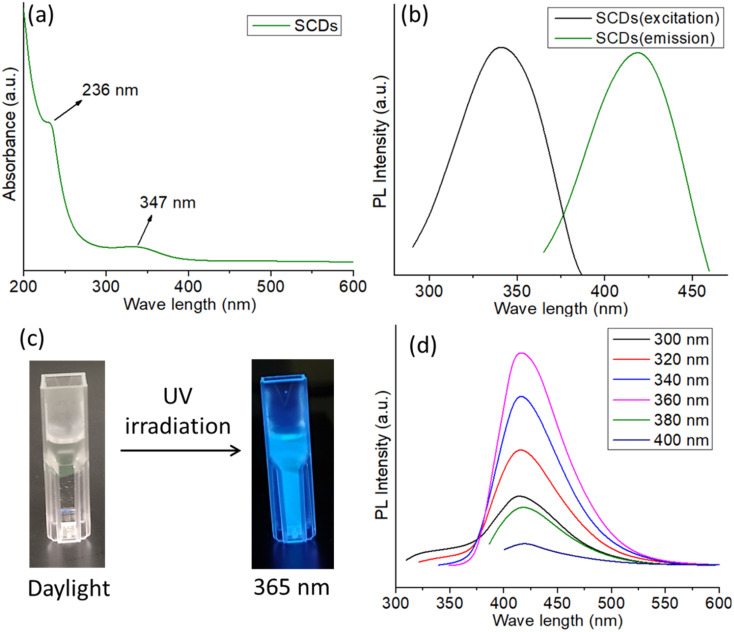
(a) UV-visible spectrum of SCDs (b) excitation and emission spectra of SCDs (c) digital images of SCDs in daylight and in 365 nm UV irradiation (d) excitation dependent PL spectra of SCDs.

The UV-visible absorption spectrum of SCDs exhibits two distinct absorption peaks, one centered at 236 nm and another at 347 nm (Fig. 2a). The peak observed at a wavelength of 236 nm can be ascribed to the π–π* transitions of the aromatic sp^2^ regions within the CDs, which suggests the existence of graphitic core structures.^32^ The absorption peak observed at 347 nm is most likely attributed to the n–π* transitions of the functional groups present on the surface of the carbon dots, such as carbonyl or amine groups. This peak indicates the existence of surface states have an impact on the optical characteristics of the SCDs. The presence of these absorption properties provides evidence that CDs have been successfully synthesized, possessing both graphitic core structures and functionalized surfaces.^38^ The excitation and emission spectra of SCDs (Fig. 2b) illustrate the photoluminescent characteristics of the CDs. When stimulated with light (usually around 360 nm), the SCDs showed an emission peak at approximately 450 nm, demonstrating blue fluorescence. The photoluminescence observed is a result of the radiative recombination of excitons, which are electron–hole pairs, within the CDs. The presence of a distinct emission peak indicates that the photoluminescent capabilities of small-sized CDs are largely influenced by both the surface states and the quantum confinement effects. The excitation spectrum provides more evidence that the most effective wavelength for achieving the highest emission is approximately 360 nm. The UV and PL spectra of CDs without sulfur doped was also carried out and the spectra were shown in Fig. S2 and S3,† respectively.

The digital images of SCDs captured under both daylight and 365 nm UV irradiation demonstrate the observable photoluminescence of the CDs as shown in Fig. 2c. In the presence of natural light, the SCDs exhibit a transparent or mildly tinted liquid state. Under the influence of 365 nm UV light, the SCDs demonstrate vivid blue fluorescence, which is easily noticeable in the photograph. The observation of photoluminescence under UV light provides additional confirmation of the existence of effective surface states and quantum confinement phenomena, which allow the CDs to generate visible light when stimulated. The PL spectra of SCDs show a significant change in the emission wavelength as the excitation wavelength varies between 320 nm and 400 nm (Fig. 2d). The observed phenomenon, referred to as a redshift, signifies that as the excitation wavelength increases, the emission shifts towards longer wavelengths. The observed behavior can be attributed to the presence of diverse surface functional groups, variations in size distribution, and the effects of quantum confinement, which are typical for CDs.^39^ The CDs' surface is abundant in functional groups, including hydroxyl, carboxyl, and amine groups. These groups generate diverse energy states and traps, which play a role in the photoluminescence phenomenon. The diverse sizes of the CDs lead to distinct band gaps, where smaller dots emit light at shorter wavelengths and larger dots emit light at longer wavelengths. In addition, the presence of defect states within the CDs can capture excitons, which in turn has a significant impact on the emission characteristics. The quantum yield of the SCDs was calculated to be 54.1%, indicating superior luminescent performance relative to previously reported quantum dots in Table S1.†

**Fig. 3:**
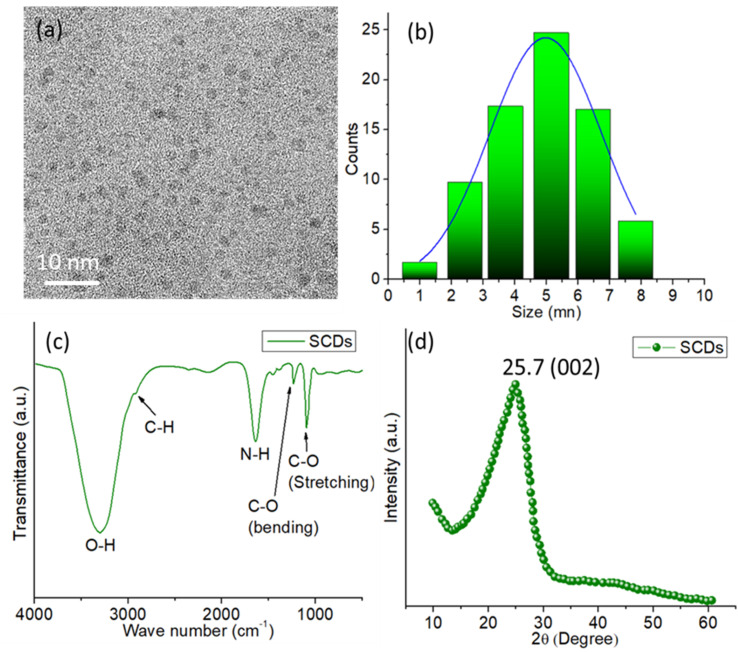
(a) TEM image of SCDs (b) particle size distribution histogram of SCDs (c) FTIR spectrum of SCDs (d) XRD pattern of SCDs.

The TEM image (Fig. 3a) demonstrates that the CDs produced are uniformly distributed and have a roughly spherical morphology. The image depicts a group of CDs, where the sizes of each individual particle are clearly discernible. The presence of a 50 nm scale bar confirms that the CDs possess a nanoscale size, falling within the range of nanometers. The TEM image exhibits a pronounced disparity in brightness, which signifies the existence of compact carbon material, distinctive of CDs. The particle size analysis of the CDs is presented in the size distribution histogram (Fig. 3b). The histogram displays a distribution that resembles a Gaussian curve, with the majority of carbon dots falling within the size range of 3 to 7 nm. The distribution's maximum value is observed at roughly 5 nm, suggesting that the carbon dots have an average size of around 5 nm. The distribution implies a relatively limited range of sizes, demonstrating consistency in the size of the produced carbon dots.

The FTIR spectrum of SCDs synthesized from sulfur and ethylenediamine using the hydrothermal technique exhibits various essential functional groups that play a vital role in determining their chemical characteristics and possible uses as shown in Fig. 3c. The presence of hydroxyl groups (–OH) on the surface of the CDs is indicated by a prominent and intense peak at around 3400 cm^−1^, which corresponds to O–H stretching vibrations.^40^ The presence of adsorbed water molecules or hydroxyl groups introduced during the synthesis process is indicated by this wide peak. The existence of aliphatic hydrocarbon chains on the surface of the carbon dots is indicated by a peak at around 2900 cm^−1^, which corresponds to C–H stretching vibrations. This suggests that the carbon dots possess aliphatic C–H bonds. Furthermore, the occurrence of a peak in the 3200–3400 cm^−1^ range can be ascribed to N–H stretching vibrations, suggesting the existence of amine groups (–NH_2_) on the surface of the carbon dots. These amine groups originate from the ethylenediamine employed in the synthesis process. The existence of a peak at around 1650 cm^−1^ indicates the stretching vibrations of carbonyl groups (C=O) on the surface of the carbon dots. This suggests that there are carbonyl or amide groups present, which might be a result of oxidation or the reaction between sulfur and ethylenediamine leading to the introduction of amide groups. The peaks detected in the range of 1000–1300 cm^−1^ are attributed to the stretching and bending vibrations of C–O bonds,^41^ which suggest the existence of several oxygen-containing functional groups, like alcohols, ethers, or carboxylic acids, on the surface of the CDs.

The XRD pattern displays a wide peak with a center at 2*θ* = 25.7°, which suggests that the SCDs have an amorphous structure (Fig. 3d). The characteristic peak observed is indicative of the (002) plane of graphitic carbon, which implies the existence of disordered or turbostratic carbon structures within the CDs. The absence of distinct diffraction peaks indicates the absence of crystalline carbon or any other crystalline contaminants in the sample. The presence of this wide diffraction peak is a distinctive feature of carbon dots, which usually comprise small, randomly aligned graphitic regions mixed with amorphous carbon. The XRD study confirms that the produced carbon dots possess an amorphous structure with characteristics resembling those of graphite.

**Fig. 4:**
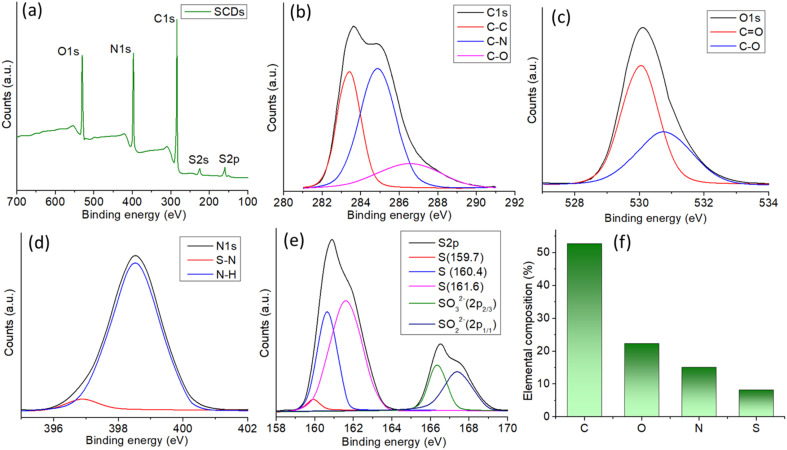
(a) XPS survey spectrum of SCDs showing all the elemental peaks. High resolution spectra of (b) C 1s (c) O 1s (d) N 1s and (e) S 2p. (f) Elemental composition obtained from the XPS.

The X-ray photoelectron spectroscopy (XPS) examination of SCDs, produced *via* a hydrothermal reaction between sulfur and ethylenediamine, offers detailed information about the elemental makeup and chemical states of the constituent elements (Fig. 4a). The XPS survey spectrum confirms the successful incorporation of carbon (C 1s), oxygen (O 1s), nitrogen (N 1s), and sulfur (S 2p) into the carbon dot matrix, as indicated by the appearance of discrete peaks corresponding to these elements. An in-depth analysis of the high-resolution spectra provides precise insights into the bonding conditions present in the SCDs. The C 1s spectrum with high resolution can be separated into four main peaks (Fig. 4b). The peak at 284.8 eV is linked to C–C bonds, indicating the presence of graphitic carbon. The peak at 285.6 eV corresponds to C–N bonds, suggesting the incorporation of nitrogen.^42^ The peak at 286.7 eV is associated with C–O bonds, indicating the presence of hydroxyl or epoxy groups. Lastly, the peak at 288.6 eV is caused by C=O bonds, implying the existence of carbonyl functionalities. The varied bonding conditions emphasize the intricate chemical composition of the SCDs. The presence of oxygenated functional groups is reinforced by the high-resolution O 1s spectra (Fig. 4c), which exhibit peaks at 531.2 eV and 532.6 eV, corresponding to C=O and C–O bonds, respectively. The N 1s spectra exhibit distinct peaks at 398.4 eV and 399.7 eV, corresponding to S–N and N–H bonds, respectively (Fig. 4d). These findings corroborate the presence of nitrogen doping and indicate potential interactions between sulfur and nitrogen in the carbon dot structure.^43^ The S 2p spectrum with high resolution shows distinct peaks at 159.7 eV and 160.4 eV, indicating the presence of elemental sulfur. Furthermore, there are further peaks observed at 161.6 eV and 163.0 eV, which correspond to the SO_3_^2−^ and SO_4_^2−^ species, respectively (Fig. 4e). The presence of sulfur-containing functional groups is expected to enhance the physicochemical features of the SCDs. The XPS data reveals that the SCDs consist of roughly 50% carbon, 25% oxygen, 15% nitrogen, and 10% sulfur in terms of their elemental composition. This composition not only verifies the effective incorporation of sulfur and nitrogen through doping but also emphasizes the notable existence of oxygenated groups, which collectively contribute to the distinctive characteristics of the SCDs (Fig. 4f). The addition of nitrogen is particularly remarkable, as it boosts the electrical characteristics of the carbon dots, potentially enhancing their effectiveness in applications such as sensing, catalysis, and bioimaging. These findings highlight the potential of SCDs in several applications, especially those that require multifunctional features derived from the multiple chemical functions contained in the carbon dot structure.

### Antioxidant behavior study

**Fig. 5:**
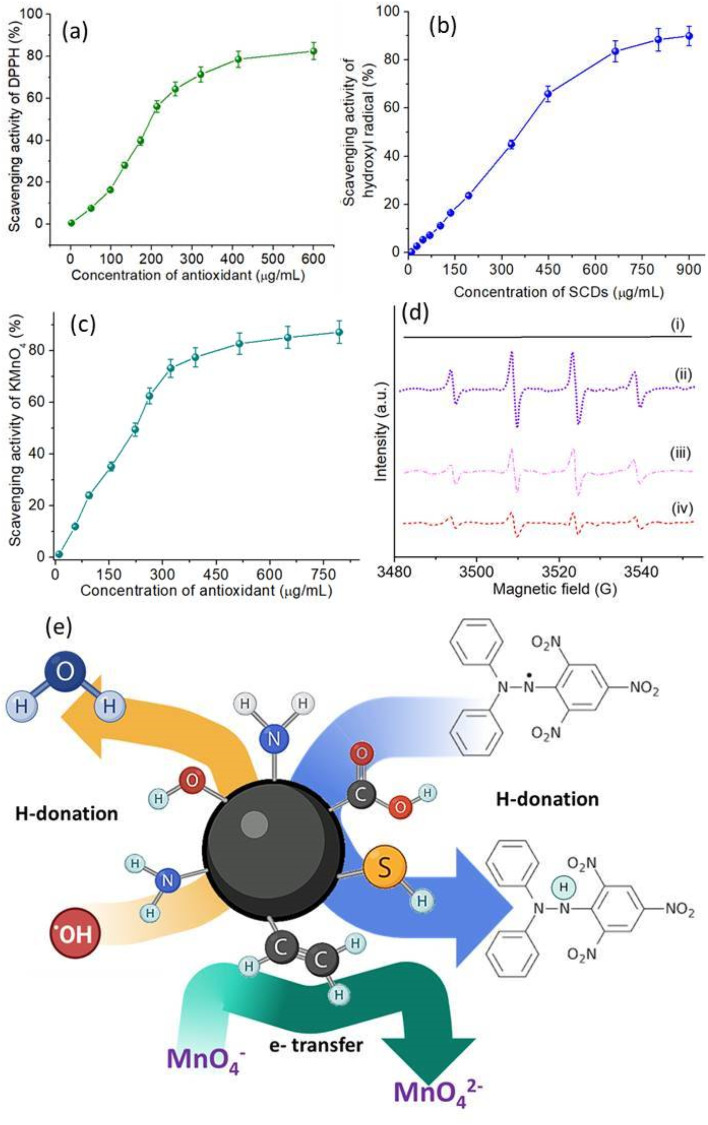
(a) The SCDs were tested for their ability to scavenge (a) DPPH radicals, (b) hydroxyl radicals, and (c) KMnO_4_ radicals (d) EPR spectra of DMPO were analyzed under various conditions. (i) DMPO alone; (ii) DMPO in combination with Fe^2+^ and H_2_O_2_; (iii) DMPO in combination with Fe^2+^ and H_2_O_2_, and SCDs at a concentration of 250 μg mL; and (iv) DMPO in combination with Fe^2+^, H_2_O_2_, and SCDs at a concentration of 450 μg mL^−1^. (e) Mechanism underlying the antioxidant activity of SCDs.

The antioxidant activity of SCDs was thoroughly examined by conducting a range of experiments that specifically targeted different types of radicals. The antioxidant capabilities of SCDs were evaluated by assessing their scavenging activity against DPPH radicals, hydroxyl radicals, and KMnO_4_ radicals, providing a thorough understanding of their antioxidant properties. Fig. 5a depicts the DPPH radical scavenging activity of SCDs at various concentrations. The data indicate that the scavenging activity increases in a manner that is directly proportional to the dose, reaching a maximum scavenging activity of around 85% when the concentration is 500 μg mL^−1^. The noteworthy DPPH radical scavenging activity indicates that SCDs have the ability to efficiently provide hydrogen atoms to counteract DPPH radicals. The concentration-dependent augmentation of the hydroxyl radical scavenging activity of SCDs is illustrated in Fig. 5b. At the maximum measured concentration of 800 μg mL^−1^, the SCDs exhibited a scavenging activity of around 80%. This suggests that SCDs have the capacity to efficiently remove extremely reactive hydroxyl radicals, which are recognized for causing significant oxidative harm in biological systems. Regarding the KMnO_4_ radical scavenging experiment shown in Fig. 5c, the SCDs exhibited strong activity, achieving a maximum scavenging rate of about 90% at a concentration of 700 μg mL^−1^. The significant degree of activity highlights the potential of SCDs in counteracting a wide range of free radicals through diverse pathways. EPR spectroscopy provided additional clarification on the antioxidant mechanism of SCDs. Fig. 5d displays the EPR spectra of DMPO (5,5-dimethyl-1-pyrroline *N*-oxide). DMPO is used to capture and detect free radicals, under various experimental settings. The DMPO spectrum alone (i) exhibits no notable signal, but the introduction of Fe^2+^ and H_2_O_2_ (ii) produces distinctive signals of hydroxyl radicals. Adding SCDs at doses of 250 μg mL^−1^ (iii) and 450 μg mL^−1^ (iv) greatly decreases these signals, suggesting that SCDs can effectively inhibit the generation of hydroxyl radicals in the Fenton reaction system.^44,45^ The shown mechanism explaining the antioxidant activity of SCDs may be observed in Fig. 5e. The mechanism entails the processes of hydrogen donation and electron transfer. SCDs can transfer hydrogen atoms to counteract the effects of free radicals, transforming them into more stable states.^46^ In addition, the inclusion of sulfur in the carbon dot structure improves the ability of electrons to move, which aids in the conversion of MnO^−^ to MnO^2−^ through reduction. The broad-spectrum antioxidant activity of SCDs is based on their ability to both donate hydrogen and transfer electrons. The EDA derived CDs were also tested for antioxidant activity having identical concentrations and condition to SCDs. The data shown in Fig. S4–S6† infer that the scavenging efficiency was much lower (50–60%) than the codoped system. Pure CDs exhibit moderate antioxidant activity owing to the presence of oxygen-containing functional groups that enable electron or hydrogen atom transfer.^47^ Nonetheless, their efficacy is frequently constrained by the insufficient diversity of active sites for radical scavenging. Single-doping with heteroatoms such as nitrogen (N) or sulfur (S) enhances antioxidant efficacy by creating supplementary active sites. Nitrogen doping can improve electron transport capabilities, hence increasing free radical scavenging efficiency. However, the existence of a singular dopant constrains the adaptability of the antioxidant systems.^33^ The co-doping of various heteroatoms, including sulfur and nitrogen, markedly improves the antioxidant capabilities of carbon dots through synergistic actions. The insertion of various functional groups results in an expanded spectrum of interactions with ROS.^48^ The incorporation of sulfur in codoped CDs imparts electron-donating properties, whilst nitrogen facilitates electron transport mechanisms. This synergism yields superior radical scavenging effectiveness relative to both pure and singly-doped CDs.

### *In vitro* drug release study

SCDs have demonstrated significant potential in drug administration, namely for the precise transportation of chemotherapeutic drugs like doxorubicin (DOX). These SCDs are generated using a hydrothermal reaction involving sulfur and ethylenediamine. This study examines the patterns of drug release from DOX-loaded SCDs under different physiological situations and analyzes the release data using several kinetic models to get insight into the underlying mechanisms.

**Fig. 6:**
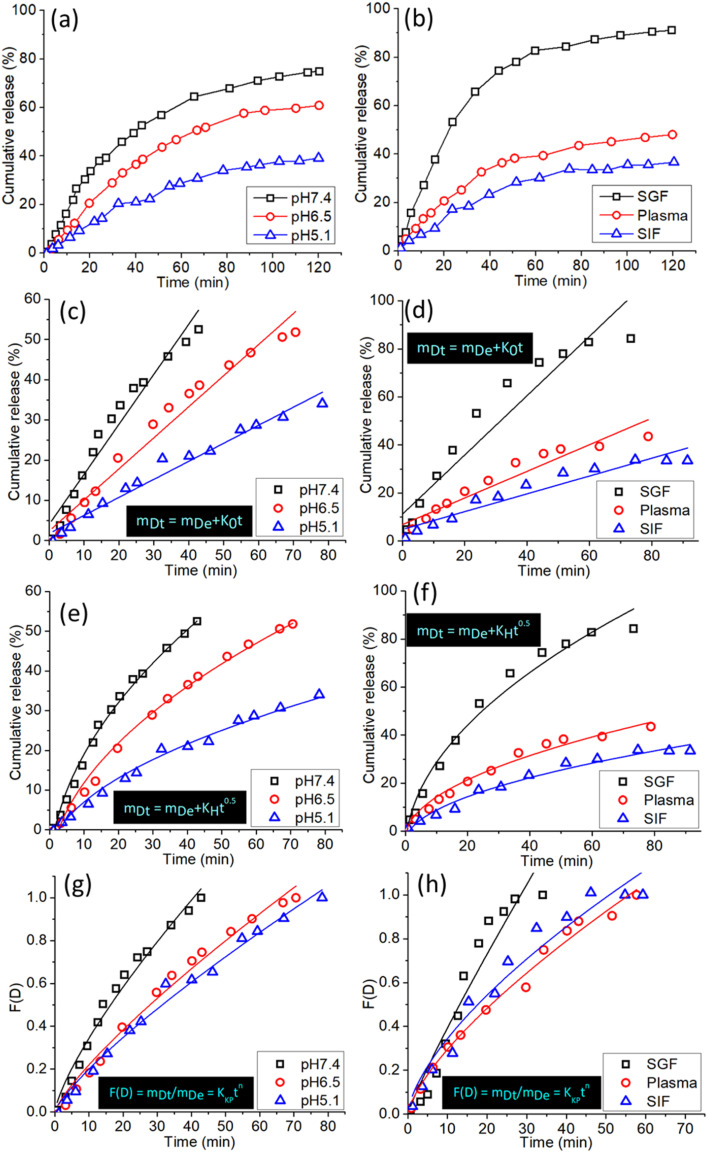
(a) Cumulative release plot of DOX from SCDs in three different pH media (b) cumulative release plot of DOX from SCDs in simulated gastric fluid (SGF), plasma, and simulated intestinal fluid (SIF). Fitting of the release data in (c) and (d) are zero-order Donbrow–Samuelov model; (e) and (f) are Higuchi model; (g) and (h) are Korsmeyer–Peppas model.

Fig. 6a displays the total release patterns of DOX from SCDs in three distinct pH conditions: acidic (pH 5.0), neutral (pH 7.4), and basic (pH 9.0). The pH of the medium has a substantial impact on the release behavior. At a pH of 5.0, there is a swift and significant liberation of DOX, with around 80% of the medication being released within the initial 24 hours. The expedited release in acidic conditions is advantageous for specific cancer treatment, as the tumor microenvironment usually has lower pH values in comparison to healthy tissues. On the other hand, the release of the substance at pH 7.4 and pH 9.0 is more regulated and slow, resulting in cumulative releases of around 50% and 40% respectively, throughout the same time frame. The pH-responsive release pattern demonstrates the potential of SCDs for precise and regulated medication delivery purposes. Fig. 6b displays the total amount of DOX released by SCDs in simulated gastric fluid (SGF), plasma, and simulated intestinal fluid (SIF). Under acidic conditions with a pH of 1.2, the release of DOX is fast, with more than 70% of the drug being released within a 24 hour period. The quick release in acidic conditions aligns with the findings observed in the pH 5.0 medium. Within a plasma environment with a pH of 7.4, approximately 55% of DOX is released over a period of 48 hours. This suggests that SCDs are capable of sustaining a protracted release of the drug in the bloodstream. Under the conditions of SIF (pH 6.8), the release is moderate, with an approximate cumulative release of 65% over the same time frame. These findings indicate that SCDs have the ability to efficiently release DOX in different bodily fluids, which makes them appropriate for delivering drugs orally or intravenously.

In order to gain a deeper comprehension of the drug release mechanism, the release data was subjected to fitting with three kinetic models: the zero-order Donbrow–Samuelov model, the Higuchi model, and the Korsmeyer–Peppas model. The zero-order model characterizes a consistent and unchanging rate of drug release over a period of time, regardless of the drug concentration. The equation for the zero-order model is:

1 *m*_Dt_ = *m*_De_ + *K*_0_*t*

The eqn (1) represents the relationship between the amount of drug released at a given time (*m*_Dt_), the initial amount of drug (*m*_De_), and the zero-order release constant (*K*_0_). Fig. 6c and d displays the alignment of the release data with the zero-order model. The plot's linearity suggests a favorable fit, indicating a consistent release rate that is desired for maintaining stable medication levels in the body.

The Higuchi model characterizes drug release as a diffusion phenomenon according to Fick's law. It is mainly suited to systems where the rate of drug release is directly related to the square root of time. The mathematical expression is:2
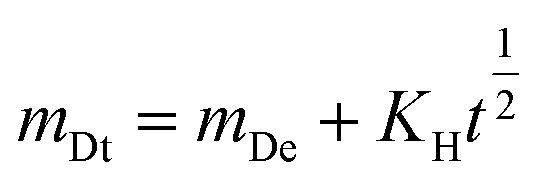


The eqn (2) the relationship between the amounts of drug released at a given time and the Higuchi dissolving constant. Fig. 6e and f displays the alignment of the release data with the Higuchi model. The linear nature of the data demonstrates that the release mechanism is diffusion-controlled, suggesting that the drug molecules diffuse through the SCDs in order to be released.

The Korsmeyer–Peppas model is employed for the analysis of drug release mechanisms from polymeric structures in cases when the release mechanism is not well-understood or is a combination of many occurrences. The mathematical expression is:3
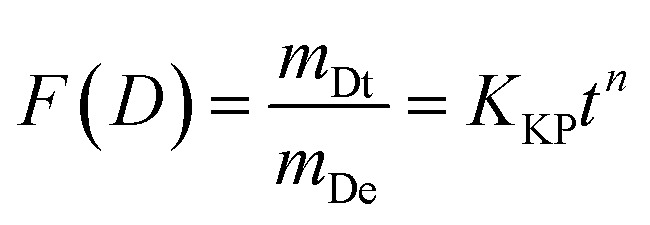


The variables in the equation are as follows: *m*_Dt_ represents the amount of drug released at a specific time ‘*t*’, *m*_De_ represents the total amount of drug released, *K*_KP_ represents the Korsmeyer–Peppas release rate constant, and ‘*n*’ represents the release exponent, which indicates the mechanism of release. Fig. 6g and h displays the alignment of the release data with the Korsmeyer-Peppas model. The values of *n* provide insights into the release mechanism: *n* ≈ 0.5 suggests Fickian diffusion, 0.5 < *n* < 1 shows anomalous transport and *n* = 1 indicates zero-order release.

The drug delivery mechanism of SCDs encompasses both pH-sensitive and diffusion-controlled release processes. In an acidic environment, the SCD matrix expands and breaks down more quickly, resulting in a fast release of DOX. This feature is beneficial for specifically targeting the acidic tumor microenvironment, guaranteeing a greater amount of the medicine is delivered to cancer cells while reducing any harmful effects on the entire body. Under neutral and basic circumstances, the discharge is more regulated and prolonged, which is advantageous for preserving therapeutic medication levels for prolonged durations. Applying the release data to the kinetic models yields additional insights. The strong correspondence with the zero-order model indicates that SCDs have the capability to maintain a consistent release rate, which is crucial for sustaining stable drug levels. The fit of the Higuchi model suggests that the release of the medication is regulated by diffusion, which is driven by the difference in concentration. The Korsmeyer–Peppas model demonstrates that the release mechanism is a blend of diffusion and erosion processes, where the release exponent *n* provides specific insights into the release dynamics. SCDs show great promise as carriers for delivering drugs, displaying pH-responsive and controlled release characteristics. Assessing the values of “*n*” under various settings can provide insights into the method by which the medication is released from the vector. The “*n*” values for anomalous diffusion from stiff non-swellable spheres are less than 0.5. In the control experiment, the models did not fit precisely because the release profile was inadequate to match the data in their individual models. Therefore, the *R*^2^ values for the control experiment were somewhat lower than the *R*^2^ values for other release data. The Korsmeyer–Peppas model provided the most accurate assessment of the drug release mechanism, unlike the other two models which did not include it. The drug release mechanism was tracked using a sequential loading and release approach.^9^ The capacity to adjust medication release according to environmental circumstances and the fitting of release data to recognized kinetic models emphasize their appropriateness for targeted and controlled drug delivery applications. These discoveries create new possibilities for utilizing SCDs in cancer treatment and other scientific fields where accurate and continuous medication administration is essential.

### Cytotoxicity assay

**Fig. 7:**
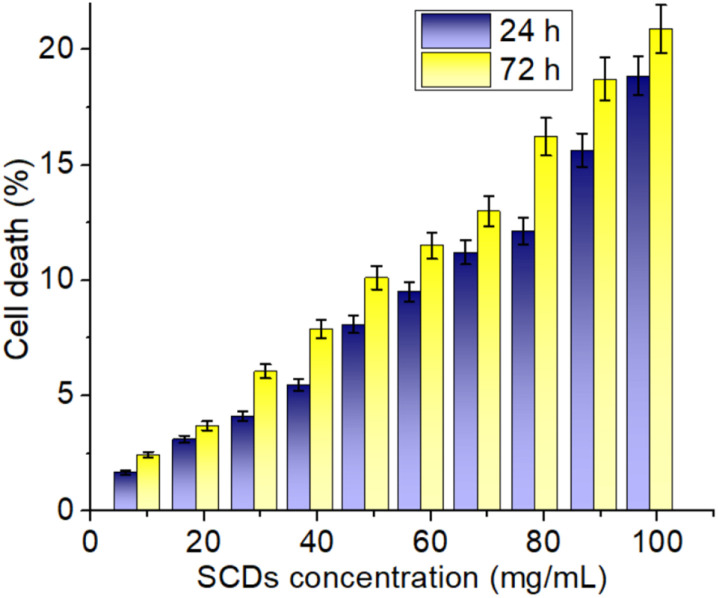
*In vitro* cytotoxicity assay of SCDs in different concentrations. The study was performed against fibroblast 3T3 cell lines.

The biocompatibility and potential for biomedical applications of sulfur-doped carbon dots (SCDs) produced from sulfur and ethylenediamine were evaluated by assessing their cytotoxicity on fibroblast 3T3 cells. Fig. 7 displays the cell viability data, specifically indicating the percentage of cell death at different concentrations of SCDs over a period of 24 and 72 hours. The findings demonstrate a distinct rise in cytotoxicity that is directly influenced by both the dosage and duration of exposure. SCDs demonstrate negligible cytotoxicity at lower dosages (10–30 mg mL^−1^), with cell mortality remaining below 10% even after 72 hours. These findings suggest that SCDs have excellent biocompatibility at lower dosages, making them well-suited for applications that require low toxicity, such as medication delivery and bioimaging. A substantial increase in cytotoxicity is found when the dose increases from 40 to 60 mg mL^−1^. The rates of cellular mortality increase to around 10–15% after 24 hours and 15–20% after 72 hours. The observed moderate cytotoxicity indicates that SCDs at these concentrations start to have more noticeable biological effects, which could be utilized for therapeutic purposes that require greater dosages to be effective. When the dose is increased to 80–100 mg mL^−1^, the cytotoxicity becomes more noticeable, resulting in cell death rates of over 20% after 72 hours. This suggests that whereas SCDs are generally not harmful at lower levels, their ability to cause cell death becomes considerable at greater concentrations. As a result, their employment may be restricted in situations where high concentrations of nanoparticles are needed. The observed rise in cytotoxicity over time is also significant. Cell death rates are much higher at 72 hours compared to 24 hours across all tested doses. This pattern indicates that extended exposure to SCDs amplifies their cytotoxic effects, highlighting the significance of this factor for long-term use. Prolonged exposure is likely to result in the accumulation of stress on the cells, potentially caused by oxidative stress, disturbance of cellular processes, or interactions with cellular components over a period of time. The observed cytotoxicity can be ascribed to multiple sources. The size and surface chemistry of SCDs are essential factors in determining how they interact with cells. SCDs' compact dimensions facilitate their effortless traversal across cellular membranes, potentially causing disruption to cellular operations. Additionally, the sulfur content in the SCDs may enhance their reactivity, resulting in the production of reactive oxygen species (ROS) that can cause oxidative stress and cell death. Furthermore, the cytotoxicity seen in a dose-dependent manner is consistent with the expected behavior of nanoparticles. Higher concentrations of nanoparticles result in more interactions with cellular components, leading to more pronounced cytotoxic effects. The MTT was also assessed without sulfur doping which showed little higher cell death as shown in Fig. S7.† The primary distinction in cytotoxicity between SCDs and carbon dots devoid of sulfur (N-CDs) resides in their engagement with cellular systems. Sulfur doping in SCDs enhances biocompatibility by altering surface chemistry and diminishing the generation of reactive oxygen species (ROS) during biological interactions. This leads to reduced cytotoxicity relative to N-CDs, which may demonstrate elevated ROS formation because to the lack of sulfur's redox balancing action. Furthermore, sulfur introduces supplementary functional groups, including thiols, which can enhance cell viability by augmenting the antioxidant capability of the dots. SCDs are more appropriate for biological applications requiring non-cytotoxic properties, such as drug delivery or bioimaging, while N-CDs may have comparatively greater cytotoxic potential.

## Conclusions

Here we detailed a one-step process for synthesizing S and N co-doped CDs that is a single-step hydrothermal process. The antioxidant behavior of SCDs was studied against DPPH, ABTS, and KMnO_4_. In all cases, the SCDs' antioxidant activity was over 80%. Furthermore, the release study of the model drug molecule used PGCDs as an example of a nano-vector. A more regulated release behavior was observed at pH 7.4 compared to pH 5.2 and 6.6, indicating that the doped PGCDs exhibited pH-responsive release behavior. The PGCDs showed no signs of cytotoxicity *in vitro*, and their ability to maintain their original shape after 24 and 72 hours proves that nanoparticles may stimulate cell growth and adhesion. One possible step toward creating a smart and adaptable material is the creation of a nontoxic fluorescent nanodot that can react to stimuli to release medications and act as an antioxidant nanomaterial.

## Data availability

Based on the request the authors will provide data.

## Author contributions

Abdullah Alarifi, and Arunkumar Thirugnanasambandam: scientific and technical discussion, Md Kasif: formal analysis, investigation, and interpretation of data. Mohd Afzal: data curation, formal analysis, investigation, writing review, and editing. Arunkumar Thirugnanasambandam: substantial contribution to conceptualization, methodology, drafting, designing, acquisition, analysis, interpretation of data, and writing – review and editing the article.

## Conflicts of interest

The authors declare no conflict of interest.

## Supplementary Material

RA-014-D4RA05994H-s001
