# Supplementary material for: N, S-codoped carbon dots for antioxidants and their nanovehicle potential as molecular cargoes
Source: RSC Adv. 2024 Oct 10;14(44):32041–52. doi: 10.1039/d4ra05994h (PMC11465998; doi:10.1039/d4ra05994h)
Supplement: RA-014-D4RA05994H-s001 [file RA-014-D4RA05994H-s001.pdf]

Supplementary Information (SI) for RSC Advances.
This journal is © The Royal Society of Chemistry 2024

N, S-codoped carbon dots for antioxidant and its nanovehicle potential as molecular cargoes

Md Kasif[a], Abdullah Alarifi[b], Mohd Afzal[b]*, Arunkumar Thirugnanasambandam[c*]

[a]*School of Energy Science and Engineering, Indian Institute of Technology Guwahati, Guwahati 781039, India*
[b]*Department of Chemistry, College of Science, King Saud University, Riyadh 11451, Saudi Arabia.*
[c]*Centre for Sustainable Materials and Surface Metamorphosis, Chennai Institute of Technology, Chennai, Tamilnadu, −600069, India.*

Email: maslam1@ksu.edu.sa (M. Afzal) and arunkumar.t@citchennai.net (A. Thirugnanasambandam)

Quantum yield measurement

The quantum yield of SCDs was calculated at an excitation wavelength of 360 nm by the following equation,[1]

$$QY = Q_R.\frac{I_{MANCDs}}{I_R}.\frac{A_R}{A_{CD}}.\frac{\eta^2_{MANCDs}}{\eta^2_R} \qquad (1)$$

Where 'Q', 'I', 'A' and 'η' represent quantum yield, intensity of luminescent spectra, absorbance at particular exited wavelength and refractive index of the solvent, respectively. The subscript 'R' and 'U' stand for the reference and unknown QY of SCDs, respectively. Quinine sulfate in 0.1 M H_2SO_4 was used as standard and its quantum yield (QY) is known to be 54% in 0.1 M H_2SO_4 solution.

Antioxidant Activity of SCDs

The free radical scavenging capacities of different concentrations of SCDs were evaluated using DPPH. Briefly, different concentrations of SCDs were introduced into a 100 µM methanolic solution of DPPH. The mixture solutions were incubated in the dark for 30 min. After 30 min in the dark, the absorbance at 515 nm was measured. The DPPH free radical scavenging was calculated by the following equation:

Scavenging activity (%) = $(A_c - A_s)/A_c$ (2)

Where A_c and A_s represent absorbance in the absence and presence of SCDs, respectively.

The scavenging of OH free radicals was examined using Fenton's reaction (Brillas et al. 2009). First, 500 µL of 1.8 mM $FeCl_2$ was added to 375 µL of an ethanolic solution containing 1.8 mM salicylic acid. The different concentrations of SCDs were added to the mixture, followed by 25

μL of 100 mM H_2O_2. After 10 minutes at 37 °C, the mixture was centrifuged at 5000 rpm for 3 minutes. Next, 150 μL of the supernatant was dispensed into a 96-well plate. The photolysis of H2O2 produced OH radicals. The absorbance at 510 nm was measured, and the scavenging activity was determined using Equation 2.

For the $KMnO_4$ reduction test, a 1 mM acidic $KMnO_4$ solution was prepared. The various concentrations of SCDs were thoroughly mixed with the $KMnO_4$ solution in a 1:3 ratio. After 20 min incubation in the dark, the mixture was centrifuged at 5000 rpm for 3 min. Next, 150 μL of the supernatant was dispensed into a 96-well plate and absorbance at 515 nm was then measured. The $KMnO_4$ radical scavenging activity was determined using Equation 2.

Table S1 Different types of doped and non-doped CDs and their quantum yield

Precursors	Doping atom	Quantum yield (%)	Ref.
Citric acid, ethylendiamine	Nitrogen	80.6	[2]
Sodium citrate, ethylendiamine	Nitrogen	21.6	[2]
Citric acid, Hexamethylenetetramine	Nitrogen	17	[3]
Glucose	-	7	[4]
m-phenylenediamine	Nitrogen	4.8	[5]
Cysteine	Nitrogen, sulfur	--	[6]
Histidine	Nitrogen	10.7	[7]
Citric acid, urea	Nitrogen	36	[8]
Acrylic acid, ethylenediamine	Nitrogen	30.5	[9]
Calcium citrate	Calcium, nitrogen	10.1	[10]
Citric acid, Triethylenetetraamine	Nitrogen	33.4	[11]
Citric acid, Triethanolamine	Nitrogen	7	[3]
Citric acid, n-heptylamine	Nitrogen	7.7	[2]
Gelatin	Nitrogen	31.6	[12]
Xylan, hydroxylamine	Nitrogen	16	[13]
Branched polyethyleneimine	Nitrogen	54.3	[14]
Biomass	---	33.3	[15]
Sulfur, ethylendiamine	Nitrogen, sulfur	54.1	This work

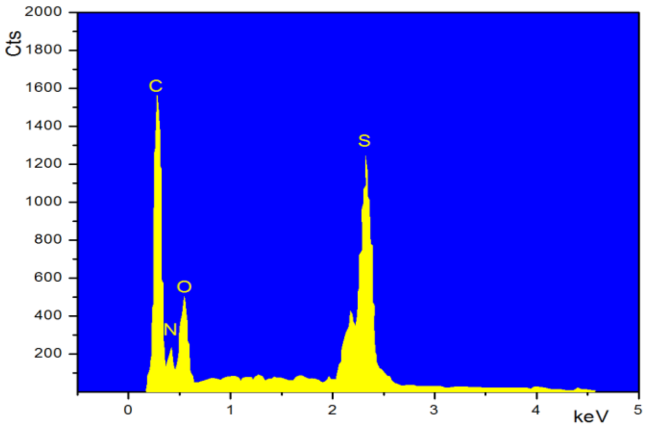

Figure S1 EDS spectrum of CDs

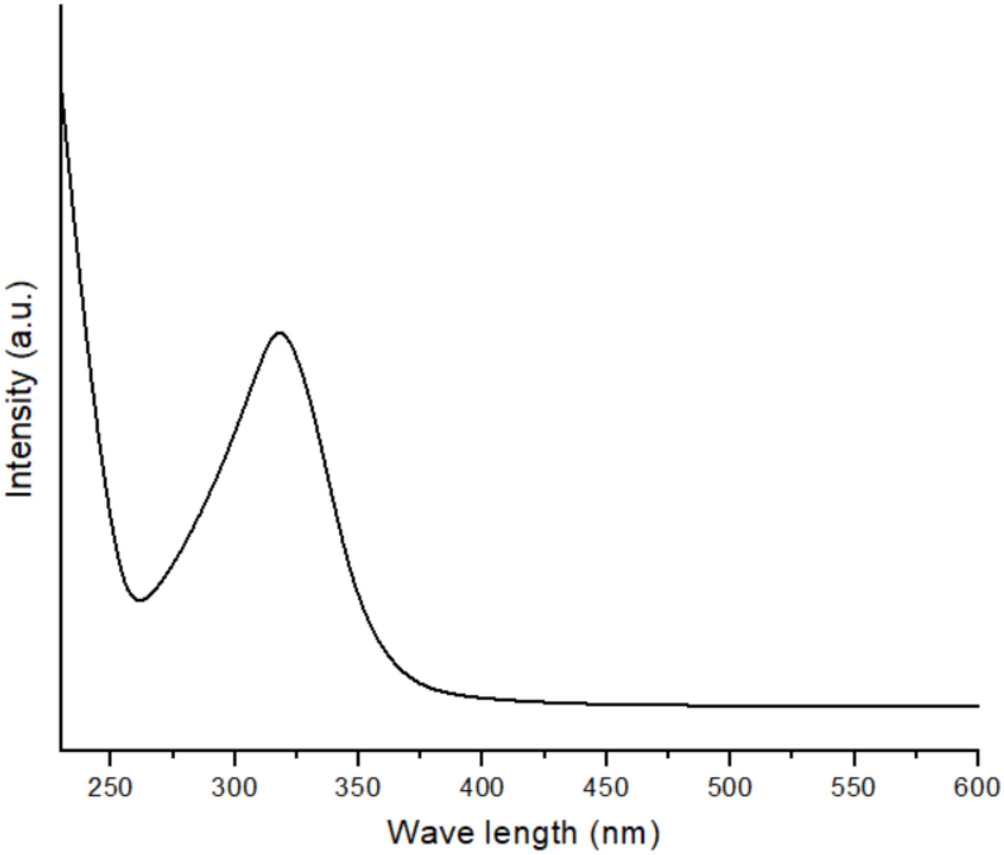

Figure S2 UV-vis spectrum of ethylene based N-doped CDs

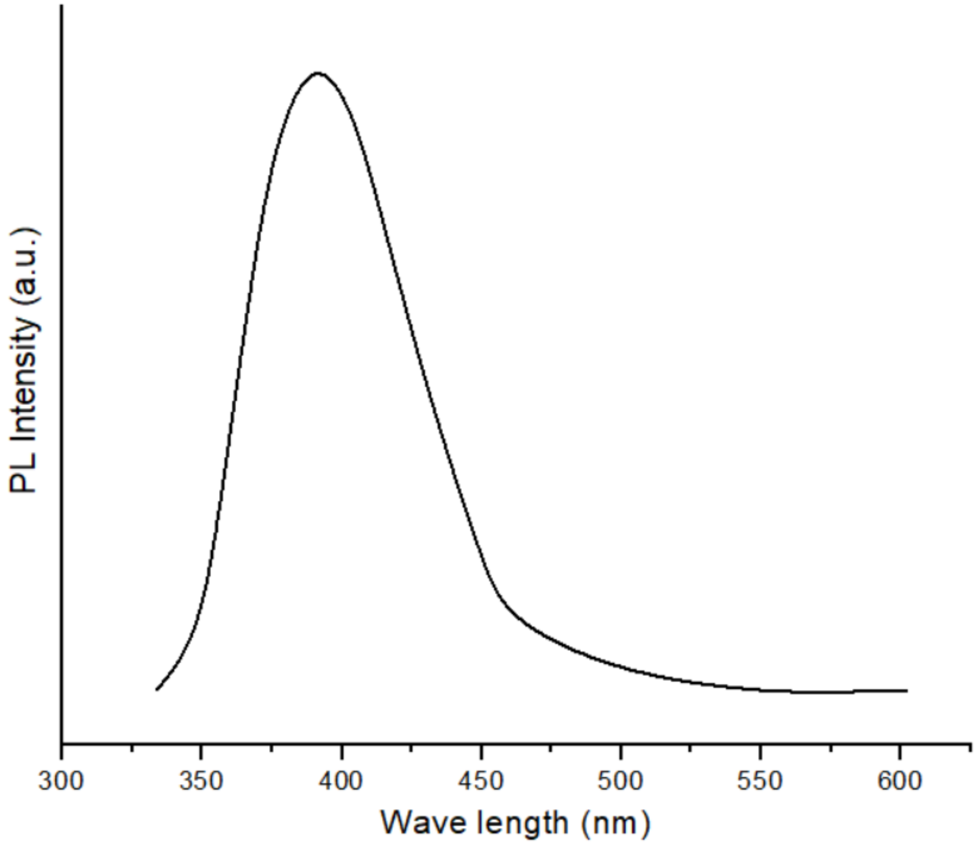

Figure S3 PL spectrum of of ethylene based N-doped CDs

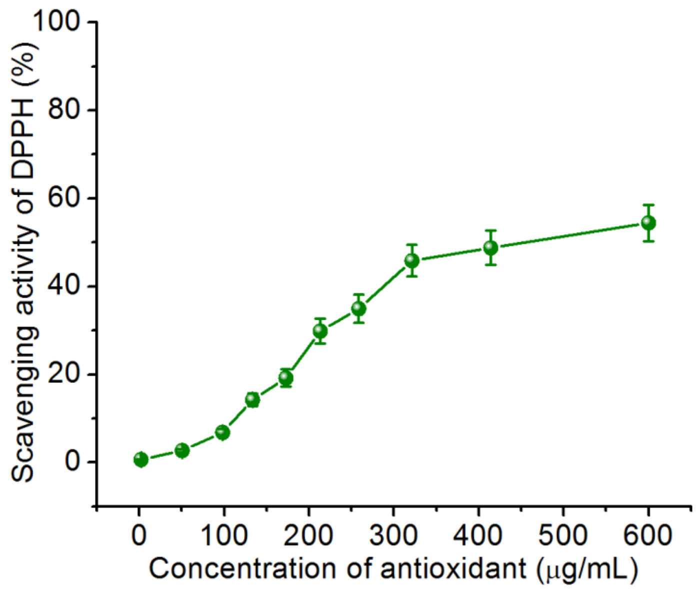

Figure S4 DPPH radicals scavenging activity of the CDs

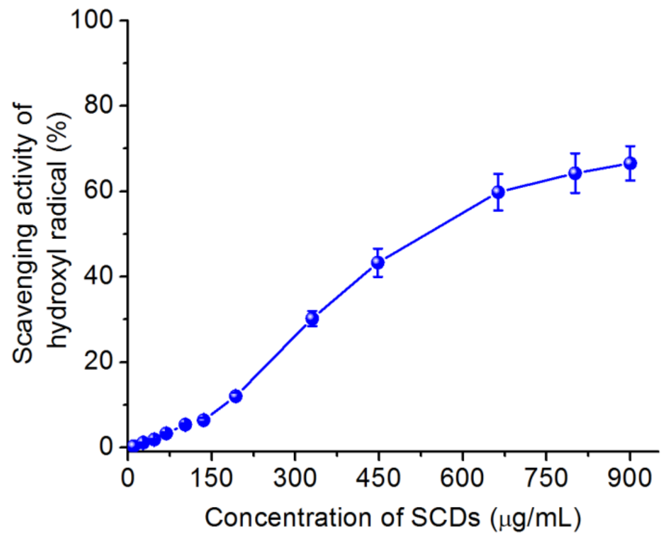

Figure S5 Hydroxyl radicals scavenging activity of the CDs

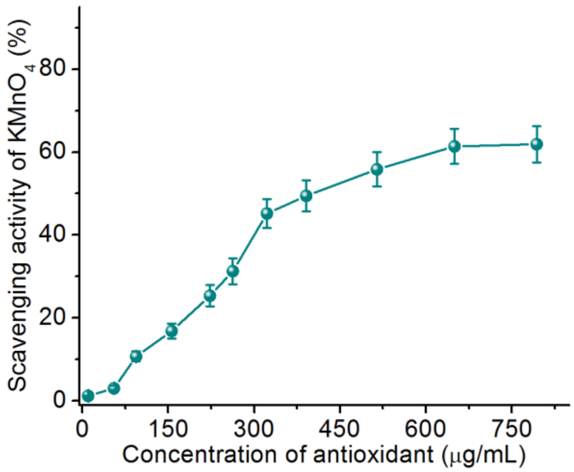

Figure S6 $KMnO_4$ radicals scavenging activity of the CDs

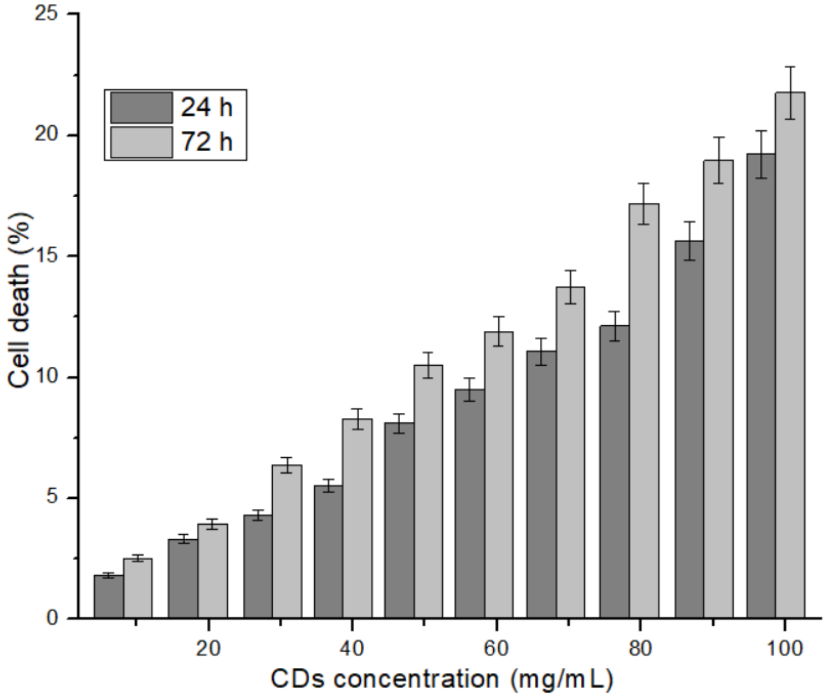

Figure S7 *In vitro* cytotoxicity assay of ethylenediamine derived CDs in different concentrations. The study was performed against fibroblast 3T3 cell lines.

References

1. Parker, C. and W. Rees, *Fluorescence spectrometry. A review.* Analyst, 1962. **87**(1031): p. 83-111.
2. Zhu, S., et al., *Highly photoluminescent carbon dots for multicolor patterning, sensors, and bioimaging.* Angewandte Chemie International Edition, 2013. **52**(14).
3. Schneider, J., et al., *Molecular fluorescence in citric acid-based carbon dots.* The Journal of Physical Chemistry C, 2017. **121**(3): p. 2014-2022.
4. Li, H., et al., *One-step ultrasonic synthesis of water-soluble carbon nanoparticles with excellent photoluminescent properties.* Carbon, 2011. **49**(2): p. 605-609.
5. Jiang, K., et al., *Red, Green, and Blue Luminescence by Carbon Dots: Full-Color Emission Tuning and Multicolor Cellular Imaging**. 2015, 1–5*.
6. Wan, J.-Y., et al., *Ionic liquid-assisted thermal decomposition synthesis of carbon dots and graphene-like carbon sheets for optoelectronic application.* RSC advances, 2016. **6**(66): p. 61292-61300.
7. Dai, H., et al., *A carbon dot based biosensor for melamine detection by fluorescence resonance energy transfer.* Sensors and Actuators B: Chemical, 2014. **202**: p. 201-208.
8. Zeng, Q., et al., *Carbon dots as a trackable drug delivery carrier for localized cancer therapy in vivo.* Journal of Materials Chemistry B, 2016. **4**(30): p. 5119-5126.
9. Zhang, P., et al., *A facile and versatile approach to biocompatible "fluorescent polymers" from polymerizable carbon nanodots.* Chemical Communications, 2012. **48**(84): p. 10431-10433.
10. Xu, M., et al., *A green heterogeneous synthesis of N-doped carbon dots and their photoluminescence applications in solid and aqueous states.* Nanoscale, 2014. **6**(17): p. 10307-10315.
11. Wang, J., et al., *High performance photoluminescent carbon dots for in vitro and in vivo bioimaging: effect of nitrogen doping ratios.* Langmuir, 2015. **31**(29): p. 8063-8073.
12. Liang, Q., et al., *Easy synthesis of highly fluorescent carbon quantum dots from gelatin and their luminescent properties and applications.* Carbon, 2013. **60**: p. 421-428.
13. Liang, Z., et al., *Sustainable carbon quantum dots from forestry and agricultural biomass with amplified photoluminescence by simple NH 4 OH passivation.* Journal of Materials Chemistry C, 2014. **2**(45): p. 9760-9766.
14. Hu, L., et al., *Multifunctional carbon dots with high quantum yield for imaging and gene delivery.* Carbon, 2014. **67**: p. 508-513.
15. Salimi Shahraki, H., A. Ahmad, and R. Bushra, *Green carbon dots with multifaceted applications–Waste to wealth strategy.* FlatChem, 2022. **31**: p. 100310.
